# Endothelial activation and injury by microparticles in patients with systemic lupus erythematosus and rheumatoid arthritis

**DOI:** 10.1186/s13075-018-1796-4

**Published:** 2019-01-23

**Authors:** Laura Atehortúa, Mauricio Rojas, Gloria Vásquez, Carlos H. Muñoz-Vahos, Adriana Vanegas-García, Rafael Andrés Posada-Duque, Diana Castaño

**Affiliations:** 1 Grupo de Inmunología Celular e Inmunogenética, Instituto de Investigaciones Médicas, Facultad de Medicina, Sede de Investigación Universitaria (SIU), Universidad de Antioquia U de A, Calle 70N 52-21, Medellín, Colombia; 2 Unidad de Citometría, Facultad de Medicina, SIU, Universidad de Antioquia U de A, Calle 70N 52-21, Medellín, Colombia; 3 Sección de Reumatología, Hospital Universitario San Vicente Fundación, Calle 64 # 51 D – 154, Medellín, Colombia; 4 Cellular and Molecular Neurobiology Area, Group of Neuroscience of Antioquia, Institute of Biology, Faculty of Exact and Natural Sciences, University of Antioquia, Medellín, Colombia

**Keywords:** Microparticles, Endothelial cells, Immune complexes, Rheumatoid arthritis, Systemic lupus erythematosus

## Abstract

**Background:**

Endothelial activation and damage is commonly observed in patients with systemic lupus erythematosus (SLE) and rheumatoid arthritis (RA) and is related to development of atherosclerosis and cardiovascular diseases. Different components of the immune system seem to participate in the endothelial injury, such as generation of autoantibodies and formation of immune complexes (ICs). Microparticles (MPs) and their immune complexes (MPs-ICs) are increased in the circulation of patients with SLE and RA; therefore, we propose these extracellular vesicles could interact and modulate the function of endothelial cells. Hence, the effect of MPs and MPs-ICs from patients with SLE and RA in endothelial cells was evaluated.

**Methods:**

Macrovascular and microvascular endothelial cells were exposed to MPs and MPs-ICs from healthy donors and patients with SLE and RA. Vesicles uptake/binding, expression of adhesion molecules, cytokine and chemokine production, monocyte adherence, and alterations of endothelial monolayer were evaluated by flow cytometry and fluorescence microscopy.

**Results:**

Endothelial cells internalized MPs and MPs-ICs and increased CD54 and CD102 expression and CCL2, CCL5, and IL-6 production after the treatment with these extracellular vesicles, which led to an increase in the adherence of classic monocytes. These vesicles also induced low expression of VE-cadherin in membrane, depolymerization of actin filaments, and formation of intercellular spaces, which led to endothelial death and increased permeability after MPs and MPs-ICs exposure.

**Conclusions:**

MPs and MPs-ICs from patients with SLE and RA increase adhesion molecules expression, chemokine production, and structural alterations in macrovascular and microvascular endothelial cells. Therefore, high counts of these vesicles in patients would promote endothelial alterations and secondary tissue leukocyte infiltration.

**Electronic supplementary material:**

The online version of this article (10.1186/s13075-018-1796-4) contains supplementary material, which is available to authorized users.

## Background

Rheumatoid arthritis (RA) and systemic lupus erythematosus (SLE) are complex autoimmune diseases with systemic inflammatory compromise, in which different organs are involved, such as kidney and joints, respectively [1–3]. Endothelial alterations of macrovasculature and microvasculature have been reported in both diseases [4]. Patients with SLE and RA, have a greater risk of developing atherosclerosis and cardiovascular diseases (CVD) than does the general population [5]. These comorbidities are due to the endothelial dysfunction of the macrovasculature in which a chronic inflammatory process is developed [5]. In addition, evident endothelial involvement of the microvasculature due to immune complex (IC) deposits has also been described, mainly in the organs where ultrafiltration processes occur [6, 7]. Despite the fact that both diseases have a similar endothelial injury pattern, evidenced by an increase in the intima-media thickness (IMT) [5] and a low response to flow motion dilation (FMD) [8], they have some differences. In SLE, a pro-coagulant tendency has been observed, associated with the presence of antiphospholipid antibodies [9]. Moreover, patients with SLE have an increased level of type 1 interferon, which might disrupt the endothelial progenitor cell activity, starting the endothelial injury [10]. Patients with RA, however, have accelerated femoral atheromatosis [11] and in those with very early RA, cardiovascular disease (CVD) has been associated with endothelial dysfunction of the coronary microvasculature [12]. The endothelial compromise also differs between SLE and RA, regarding which organs are mainly affected by microvasculature injury; patients with SLE have renal vascular lesions such as thrombotic microangiopathy, lupus vasculopathy, vascular immune deposits, and arterial sclerosis [13], whereas patients with RA have synovial vascularization in the joints, characterized by vascular congestion, edema, and cellular infiltration [14].

Endothelial activation and injury in SLE and RA have been characterized by an increase in the expression of adhesion molecules, production of pro-inflammatory cytokines and pro-thrombotic factors, oxidative stress upregulation, and abnormal vascular tone modulation [4]. These tissue alterations apparently involve cell death and increase the permeability of the endothelial monolayer [15]. Endothelial activation through toll-like receptor ligands promotes recruitment of leukocytes, such as monocytes and neutrophils, which have been directly implicated in endothelial injury [16]. However, humoral immune response dysregulation, which is a hallmark of these systemic autoimmune diseases, also participates in endothelial dysfunction by producing autoantibodies that form ICs with autoantigens, either in soluble form or as a part of vesicular structures, such as microparticles (MPs) and apoptotic bodies [17, 18].

MPs are small heterogeneous extracellular vesicles released by a variety of cell types under physiological conditions and after activation, injury, and apoptosis [19, 20]. MPs participate in the intercellular exchange of information because they carry proteins, nucleic acids, receptors, and other macromolecules from their parental cells [21, 22]. Thus, these vesicles are recognized as biological effectors in inflammation, angiogenesis, vascular injury, and thrombosis [23]. Additionally, MPs seem to participate in the pathogenesis of RA and SLE, as they are increased in circulation [17, 24], are a good source of alarmins and autoantigens, and form ICs (MPs-ICs) in these patients [25, 26]. Therefore, we propose that the increase in the amounts of MPs and MPs-ICs observed in the circulation of these patients might have a deleterious effect on the endothelium and can contribute to the activation and injury of these tissues in RA and SLE. In fact, Marcos-Ramiro et al*.* in 2014 [27] and Edrissi et al*.* in 2016 [28] reported that MPs from different sources reduced transendothelial resistance (TEER) of endothelial monolayers.

Considering this information, the effects of MPs and MPs-ICs from patients with RA and SLE, on microvascular and macrovascular endothelial cells were evaluated in this study. Our results showed that MPs and MPs-ICs from patients with RA and SLE induced activation and injury of endothelial cells of macrovasculature and microvasculature in a dose-dependent manner.

## Methods

### Sample collection, MP isolation, and opsonization

Venous blood was collected from nine patients with SLE, nine patients with RA, and six healthy controls (HCs) in Vacutainer collection tubes containing sodium citrate (0.109 M, BD Vacutainer, Franklin Lakes, NJ, USA). Patients were recruited at the Rheumatology Service of “Hospital Universitario San Vicente Fundación” (HUSVF, Medellin, Colombia) and were diagnosed according to the American College of Rheumatology (ACR) criteria, revised in 1997 for SLE [29] and the European League Against Rheumatism/ACR 2010 for RA [30]. All patients with RA and eight patients with SLE were women. The median and age range of the patients with SLE were 26 (18–39) years, six patients had active SLE (aSLE), and three patients had inactive (iSLE) disease, defined according to the systemic lupus erythematosus disease activity index selena modification, in iSLE < 4 or aSLE ≥ 4 [31]. For patients with RA, the median and age range were 56 (39–66) years, three patients had active (aRA) and six patients had inactive (iRA) disease; they were classified according to the Disease Activity Score 28 (in iRA < 2.6 or aRA ≥ 2.6) [32]. The patients with SLE [33] and RA [34] included in this study belong to previously published cohorts, in which MPs were characterized in detail. As HCs, we included six women of similar ages according to the median age of each study group.

MPs were isolated from platelet-poor plasma (PPP) and were opsonized as we previously described [33]. Briefly, blood samples were centrifuged immediately after collection at 1.800 *g* for 10 min at 21 °C to separate plasma. PPP was obtained from plasma at 3.000 *g* for 20 min at 21 °C, and this last fraction was additionally centrifuged at 16.900 *g* for 1 h at 21 °C to enrich MPs. The MP pellets were immediately frozen in filtered phosphate-buffered saline (PBS; Gibco, New York, NY, USA) at − 70 °C until use. Some MPs were opsonized with purified immunoglobulin G (IgG) (MPs-ICs) from patients, for 1 h at 37 °C (Additional file 1: Figure S1A). Total IgG was previously obtained from serum samples taken from 16 seropositive patients with SLE (with high levels of antinuclear antibodies (ANAs), anti-DNA and/or anti-Smith) and 16 seropositive patients with RA (with high levels of anti-cyclic citrullinated peptides antibodies (anti-CCP)) using a NAb™ Protein G Spin Kit (Thermo scientific, Waltham, MA, USA) according to the manufacturer’s instructions. Protein concentration was quantified using the bicinchoninic acid assay (BCA; Thermo Fisher Scientific Inc). IgG enrichment was verified by protein electrophoresis with silver staining and western blot (data not shown). The final IgG preparation used for opsonization from patients with SLE contained 1:1280 ANAs (mottled pattern, indirect immunofluorescence (IIF) using HEP-2 cells), 1:40 anti-DNA (IIF), 1220 units anti-Smith (ELISA), 1270 units anti-Ro/SSa (ELISA), 90 units anti-La/SSb (ELISA), and 7630 units anti-ribonucleoprotein (RNP, ELISA). The final IgG preparation used for opsonization from patients with RA contained 286.3 units anti-CCP (CCP3 IgG ELISA). All these kits were purchased from Inova (San Diego, CA, USA).

MPs and MPs-ICs from three different controls and patients in each study group were mixed to constitute a pool. MPs-ICs pools were those that formed ≥ 28% of ICs in patients with RA and ≥ 38% in patients with SLE; MP pools were those that formed < 6% of ICs in controls and patients (Additional file 1: Figure S1B). These thresholds were established according to the distribution of the circulating MPs-ICs frequency in a population of patients with SLE [33] and RA [34], which was previously studied by us (for a detailed explanation of this analysis, please refer to the legend of Additional file 1: Figure S1C). Each pool was counted by flow cytometry as we previously described [33]. Three different pools were evaluated in this study in the case of patients with SLE and RA, and two pools for HCs; these pools induced similar responses inside each study group (Additional file 2: Figure S2).

### Endothelial cells

Human umbilical vein endothelial cells (HUVEC), human dermal microvascular endothelial cells (HMVEC-D), and human lung microvascular endothelial cells (HMVEC-L) were purchased from Lonza-Clonetics (Brasilea, Suiza) and grown in endothelial basal medium (EBM-2) supplemented with endothelial cell growth supplement (EGM®-2 MV BulletKit®) according to the manufacturer’s instructions. The stability of endothelial cells was confirmed by staining with anti-CD105-APC (clone 43A3), anti-CD31-PE (clone WM59), and anti-VWF-FITC (Biolegend, San Diego, CA, USA) (Additional file 3: Figure S3). Therefore, a maximum of five subcultures in each type of endothelial cell was used.

### MP uptake/binding assays and acid compartment measurement

MPs and MPs-ICs from patients with RA were stained with 3 μM carboxyfluorescein succinimidyl ester (CFSE; Thermo Scientific) and then washed twice with PBS. HUVEC were placed in contact with labeled vesicles (at a proportion 1:3 cells to vesicles), centrifuged at 700 *g* for 1 min, and incubated for 1 h at 37 °C. Cells were washed, detached, and a first acquisition was performed on the flow cytometer to estimate the frequency of endothelial cells that bind and uptake these vesicles, then 0.04% trypan blue (Sigma-Aldrich, St Louis, MO, USA) was added to quench the extracellular fluorescence of bound and non-internalized MPs, and a second acquisition was performed.

For some experiments, after treating HUVEC with labeled (5 μM CFSE) and unlabeled MPs and MPs-ICs for 1 and 24 h, cells were incubated with 0.1 μM or 2.5 μM LysoTracker Red DND-99 acid tropic probe (Invitrogen, Carlsbad, CA, USA) for 2 h at 37 °C; these cells were washed and analyzed by flow cytometry and epifluorescence microscopy, respectively. For this last case, fluorescence profiles of CFSE and LysoTracker were obtained using Image Pro Plus software (Media Cybernetics, Washington, MD, USA) and cells were selected using the region of interest (ROI) tool.

### Endothelial cell culture with MPs and MPs-ICs

HUVEC, HMVEC-L, and HMVEC-D were grown to confluence between 80% and 90%; thereafter, these cells were cultured alone or with MPs and MPs-ICs from patients with SLE and RA and MPs from HCs at a proportion of 1:3 for 24 h in EBM-2-supplemented medium at 37 °C and 5% CO_2_. As a positive control for activation, these cells were treated with 100 ng/mL lipopolysaccharide (LPS; *Escherichia coli* 026:B6) (Additional file 4: Figure S4A). Supernatants were collected and frozen at − 20 °C until cytokine and chemokine levels were measured. Endothelial cells were harvested for flow cytometry analysis as explained later.

Endothelial cells were blocked (0.01% sodium azide, 10% fetal bovine serum (FBS), and 1% bovine serum albumin (BSA); all from Sigma-Aldrich) for 10 min at 4 °C. Cells were simultaneously stained with anti-CD102-PE (clone CBR-IC2/2), anti-CD54-Pacific Blue (clone HA58), and anti-HLA-DR-APC-Cy7 (clone L243) (all from Biolegend) for 20 min at 4 °C in the dark. In addition, endothelial cells were independently stained with annexin V-FITC and propium iodide (PI) (BD Pharmingen™, Franklin Lakes, NJ, USA) in the presence of annexin binding buffer (BD Pharmingen™) for 15 min at 4 °C to assess the phosphatidylserine exposure and permeability of the plasma membrane, respectively. Samples were immediately acquired (10,000 events) using an LSR Fortessa flow cytometer with the FACS DIVA software (BD Biosciences, San José, CA, USA). Data were analyzed using FlowJo (Version 7.6.2, FLOWJO, LLC. Ashland, OR, USA) software; the fluorescence minus one method was performed for each antibody to determine the positive and negative events [35].

### Cytokine and chemokine measurement

Supernatants were incubated with capture beads to evaluate the concentrations of IL-6, IL-8, IL-10, IL-12p70, IL-1β, and TNF-α using a Human Inflammatory Cytokines CBA kit (Cytometric Bead Array, BD Biosciences) according to the manufacturer’s instructions. To detect the production of chemokines, Human CCL3 and CCL2 ELISA Ready-SET-Go (eBioscience, Waltham, MA, USA), RANTES (CCL5) Human ELISA Kit (Abcam, Cambridge, MA, USA), and CX3CL1 Human ELISA Kit (Thermo Scientific, Waltham, MA, USA) were used following manufacturer’s instructions. Cytokine concentrations were obtained using the standard curve of each respective kit.

### Monocyte adhesion to endothelial cells

HUVEC and HMVEC-L were cultured with MPs and MPs-ICs from patients with SLE and RA for 24 h as previously stated. Next, the cells were washed with EBM-2 to remove excess vesicles. Monocytes were isolated from 50 mL of venous blood from healthy individuals using RosetteSep™ Human Monocyte Enrichment Cocktail (STEMCELL Technologies) according to the manufacturer’s instructions. Total monocytes (purity > 90%) were stained with 0.2 μM CFSE (Thermo Scientific) for 45 min at 37 °C, washed twice with PBS, and stained with CD14-RD1 (clone 322A-1; Beckman Coulter, Brea, CA, USA) and CD16-FITC (clone 3G8; BD Pharmigen™) antibodies for 15 min at 4 °C. Classical (CD16-, purity > 95%) and non-classical (CD16+, purity > 90%) monocytes were sorted by using MoFlo™ XDP (Beckman Coulter). CD16- and CD16+ monocytes were added to endothelial cells at a proportion 1:1, centrifuged at 200 *g* for 1 min, and cultured for 30 min at 37 °C. Non-adherent monocytes were removed by multiple washes with EBM-2. Adherent monocytes were visualized and counted in an inverted epifluorescence microscope (Nikon ECLIPSE TS 100; Nikon, Tokyo, Japan) using a ×40 objective in at least five different fields per experimental condition.

### Evaluation of endothelial monolayer integrity

HUVEC and HMVEC-L were grown to confluence on sterile glass slides of 12 mm (Marienfeld, Lauda-Königshofen) that were previously covered with 4% gelatin (Sigma-Aldrich) for 40 min at 37 °C. Cells were treated for 24 h with MPs and MPs-ICs from patients with SLE and RA and also with MPs from HCs at a proportion 1:3, as previously stated. Then, the cells were fixed in two steps: first 1% paraformaldehyde (PFA) and second 4% PFA for 5 min at 37 °C each time; cells were washed with CBS 1X ((10 mM 2-(N-Morpholino) ethanesulfonic acid, Sigma-Aldrich), 138 mM KCl (Merck, Darmstadt, Alemania), 3 mM MgCl_2_ (Sigma-Aldrich), 2 mM ethylene glycol-bis(β-aminoethyl ether)-N,N,N′,N′-tetraacetic acid (VWR, Pensilvania, Radnor), and 0.32 M saccharose (Sigma-Aldrich)). Cells were incubated with 50 mM NH_4_Cl for 10 min, permeabilized with 0.25% Triton X-100 for 5 min and blocked with 2% FBS for 1 h at room temperature. Primary mouse monoclonal antibody against human VE-cadherin (1:2000; R&D Systems, Minneapolis, MN, USA) was added and incubated overnight at 4 °C. Polyclonal secondary goat against mouse IgG (1:2500; Jackson ImmunoResearch Laboratories, Inc., West Grove, PA, USA), Phalloidin-Alexa Fluor 488 (1:1000; Thermo Scientific), and Hoechst 33342 (1:5000; Invitrogen) were added for 1 h at room temperature. Cells were preserved with FluorSave ™ solution (Calbiochem, Washington, DC, USA) on glass slides.

Samples were analyzed in an inverted microscope of epifluorescence (IX 81; Olympus, Tokyo, Japan) using Image Pro Plus software. Sequential images of the same focal plane were acquired using a CCD camera cooled − 20 °C (Hamamatsu Photonics, Japan) for ≥ 5 different fields per sample and ×20 and ×60 objectives with a numerical aperture of 0.45 and 1.45, respectively. The data are presented as a description of the images and determining the area and perimeter of the spaces in which the unions between endothelial cells were lost (GAPs); the GAPs were manually delineated using ImageJ 1.5 software (WS Rasband, National Institute of Health. Bethesda, MD, USA, https://imagej.nih.gov/ij/). Nuclei were automatically delineated using the same software and were considered fragmented and condensed, with a diameter < 14 μm. In addition, fluorescence profiles were obtained for the expression of VE-cadherin, F-actin, and Hoechst 33342 using Image Pro Plus software and cells were selected using the ROI tool.

### Endothelial permeability assays

HUVEC (5 × 10^4^ cells) were seeded and grown for 5 days on Transwells® with 0.1-μm pores and 24-mm diameters (Corning Costar, New York, NY, USA) that were previously covered with 4% gelatin for 40 min at 37 °C. Then, the cells were cultured with MPs and MPs-ICs from patients with SLE and RA for 24 h. Dextran-FITC (500 μg/mL, 4000 kDa; Sigma-Aldrich) was added to the upper chamber and incubated for 2 h at 37 °C. Subsequently, the medium in the lower chamber was homogenized and the fluorescence signal in 100 μL of medium was evaluated using a fluorometer (Varioskan TM LUX multimode microplate reader; Thermo Scientific). The data were normalized through a percentage of permeability that was calculated according to the following equation: % of permeability = (FITC fluorescence of the sample/FITC fluorescence of Transwells without cells) × 100%.

### Statistical analysis

Expression of membrane molecule, chemokine, and cytokine concentrations, and the number of adhered monocytes to endothelium among cells treated with and without MPs and MPs-ICs were compared using two-way analysis of variance (ANOVA) (data are presented as the mean ± standard deviation (SD)). GAP areas and perimeters, percentages of condensed and fragmented nuclei, and percentages of endothelial permeability among the study groups were compared using the Kruskal–Wallis test and Dunn’s post hoc test (data are presented as the median ± interquartile range). Percentages of uptake and binding were compared using the Mann–Whitney test. LysoTracker signals were compared between cells treated and untreated with MPs and MPs-ICs using the Wilcoxon test, and for microscopy analysis, the correlation between green and red fluorescences was determined by Pearson correlation coefficient analysis. Statistical significance was set at the critical values of *p* ≤ 0.05 (*), *p* ≤ 0.01 (**), and *p* ≤ 0.001 (***). The analyses were performed using GraphPad Prism 6 (GraphPad Software, Inc., La Jolla, CA, USA) and in some cases using StatGraphics Centurion XVI software (StatPoint Technologies, VA, USA).

## Results

### Endothelial cells internalized MPs, and MPs-ICs from patients with SLE and RA

**Fig. 1:**
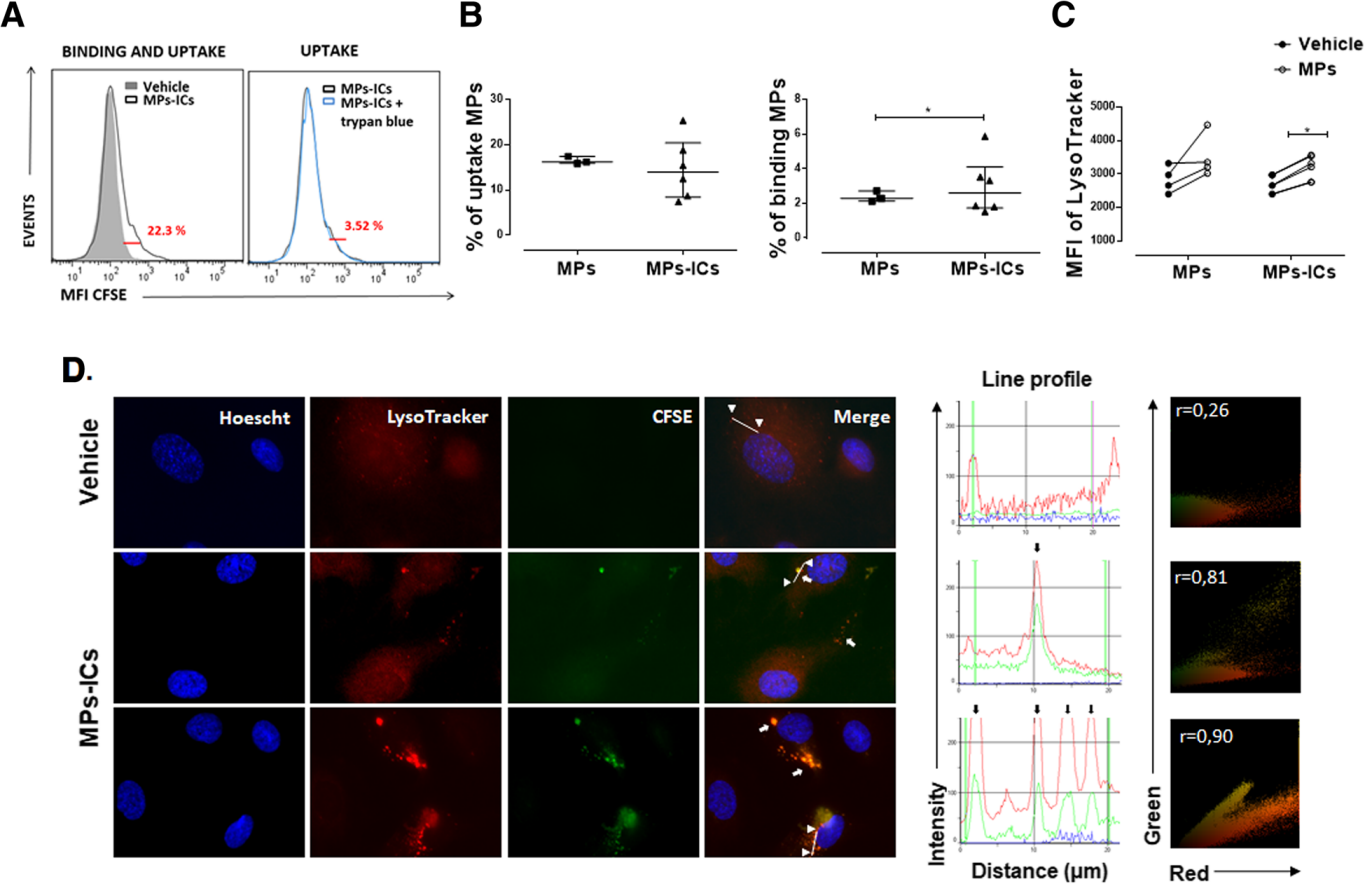
Human umbilical vein endothelial cells (HUVEC) internalize microparticles (MPs) and microparticles that form immune complexes (MPs-ICs). **a** Representative Overton subtraction analysis that shows the binding and uptake (left, 22.3%) of MPs-ICs previously labeled with carboxyfluorescein succinimidyl ester (CFSE) to HUVEC; filled gray, cells without MPs-ICs (vehicle); black line, cells with MPs-ICs. Right, percentage of binding and uptake of MPs-ICs (22.3%) with (blue line) and without trypan blue; the difference between these histograms shows the percentage of binding (3.52%). **b** Percentage of cells that take up (left) and percentage of cells that bind (right) MPs and MPs-ICs. Data are presented as the median ± interquartile range. Mann–Whitney test, *p ≤ 0.05, *n* = 3–6. **c** Mean fluorescence intensities (MFI) of LysoTracker in HUVEC without treatment (vehicle) and treated with MPs and MPs-ICs for 24 h and acquired by flow cytometry. Wilcoxon test, *p ≤ 0.05, *n* = 4. **d** Fluorescence profile (middle) and representative images (left) of the fluorescent labeling of the nucleus (blue), acid compartments (red), MPs-ICs (green), and the superposition of these images (merge) for HUVEC without treatment (vehicle) and treated with MPs-ICs from patients with rheumatoid arthritis (RA) over 1 h. White arrows indicate the presence of MPs-ICs and acid compartments, head arrows show the points from which the line was drawn (region of interest (ROI)) to determine the fluorescence profile, and black arrows indicate the peaks of colocalization between red and green. Right, Pearson correlation coefficient (*r*) of the same ROI is shown

MPs participate in cellular communication and can contact target cells by different mechanisms [22, 36–38]. For this reason, the capacity of endothelial cells to bind and take up MPs and MPs-ICs from patients with RA and SLE was evaluated. To assess this, fluorescent-labeled vesicles from patients with RA were used. As shown in Fig. 1a and b, between 8% and 26% of the endothelial cells internalized MPs and MPs-ICs; a smaller proportion of these cells kept these vesicular structures on their surface (between 2% and 5%) at the time evaluated (Fig. 1b). Endothelial cells bound more MPs-ICs than MPs (Fig. 1b). Incorporation of the acid tropic probe LysoTracker by endothelial cells after treatment with MPs and MPs-ICs was observed as an indirect indicator of endocytosis. Both kinds of vesicles induced an increase in the fluorescent signal of this probe in HUVEC, but the increase was only significant for MPs-ICs (Fig. 1c). To confirm these results, the internalization of fluorescent-labeled vesicles and incorporation of LysoTracker by endothelial cells were evaluated by fluorescence microscopy, which demonstrated that MPs-ICs were found in cytosol together with acid compartments at the same place (white arrows, Fig. 1d). The fluorescence profile and Pearson correlation confirmed the colocalization between MPs-ICs (green) and acid compartments (red) (Fig. 1d). These findings suggest that endothelial cells internalized MPs and MPs-ICs by an endocytic pathway that seems to allow acidification of these compartments.

### MPs and MPs-ICs from patients with SLE and RA activated endothelial cells from macrovasculature and microvasculature

**Fig. 2:**
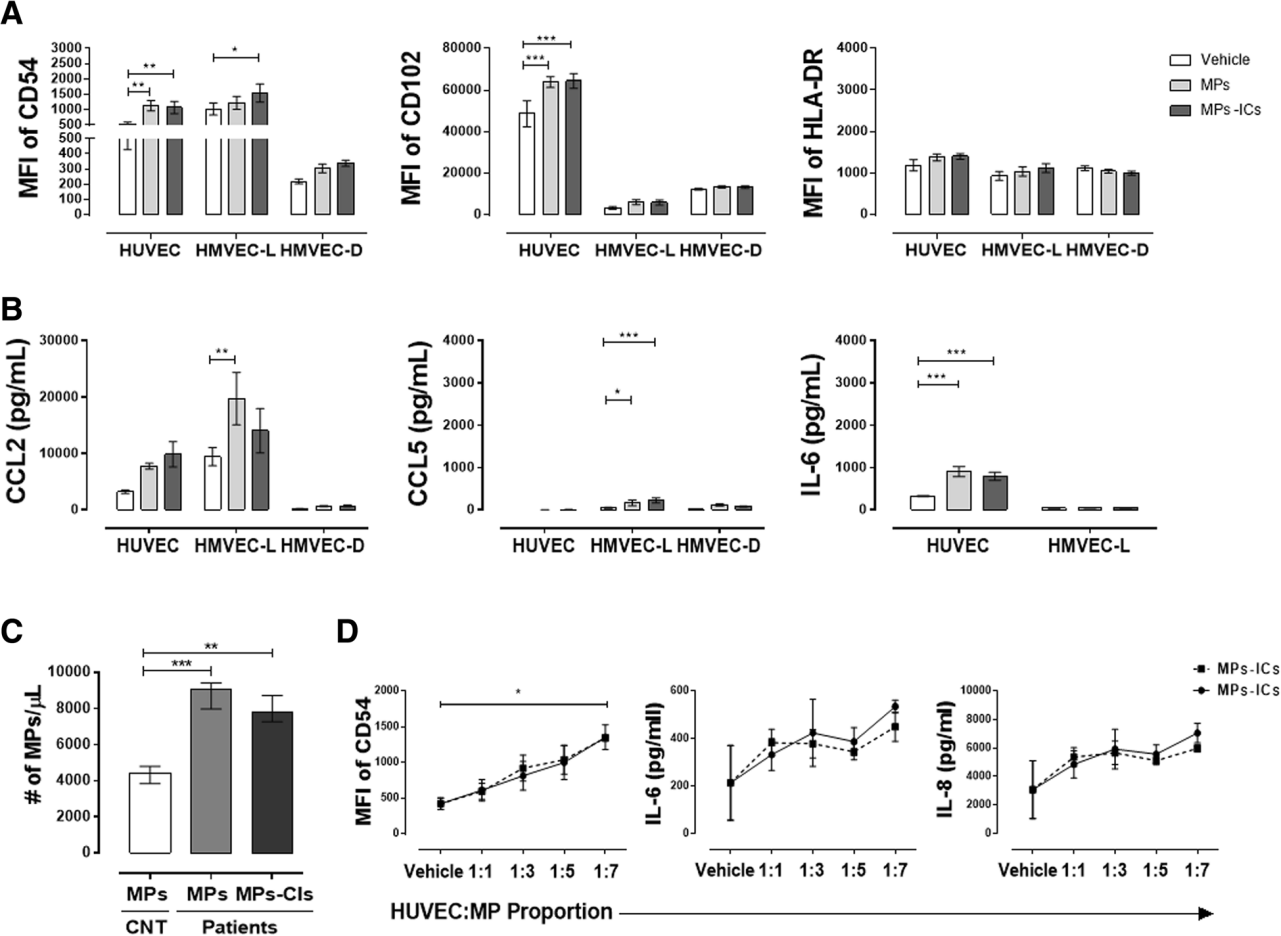
Microparticles (MPs) and microparticles that form immune complexes (MPs-ICs) from patients with systemic lupus erythematosus (SLE) and rheumatoid arthritis (RA) increased the expression of adhesion molecules and soluble factors in endothelial cells. **a** Mean fluorescence intensity (MFI) of CD54, CD102, and HLA-DR in endothelial cells of the macrovasculature (human umbilical vein endothelial cells (HUVEC)) and microvasculature (human lung microvascular endothelial cells (HMVEC-L) and human dermal microvascular endothelial cells (HMVEC-D)) without treatment (vehicle) and treated with MPs and MPs-ICs from patients with RA and SLE for 24 h. Data are presented as the mean ± SD. Two-way analysis of variance, *p ≤ 0.05, f2:8 **p < 0.01, and ***p < 0.001, *n* = 6–8. Accumulation of the chemokines **b** CCL2, CCL5, and the cytokine IL-6 in supernatants of HUVEC, HMVEC-L, and HMVEC-D treated with MPs and MPs-ICs. The white bar corresponds to cells without treatment (vehicle); the light gray bar corresponds to cells treated with MPs, and the bar corresponds to cells treated with MPs-ICs. Data are presented as the mean ± SD. Two-way analysis of variance, *p ≤ 0.05, **p < 0.01, and ***p < 0.001, *n* = 6–8. **c** Number of MPs and MPs-ICs per microliter obtained from healthy controls (HCs) and patients with RA and SLE and counted by flow cytometry. **d** MFI of CD54, levels of IL-6 and IL-8 in supernatants of HUVEC treated with different proportions (1:1, 1:3, 1:5, 1:7) of MPs (continuous black line) and MPs-ICs (broken black line) from patients with RA. Data are presented as the median ± interquartile range. Kruskal–Wallis, *p ≤ 0.05, **p < 0.01, and ***p < 0.001, *n* = 3–6

Endothelial cells are very heterogeneous, have specialized roles in different locations, and vary in responses to stimuli, injury, and repair [39, 40]. Considering this and the fact that patients with RA and SLE have an increase in circulating MPs [24, 33], especially those that form immune complexes (MPs-ICs) [25, 41], the response of macrovascular and microvascular endothelial cells to these vesicles was evaluated. The treatment of macrovascular HUVEC with MPs and MPs-ICs induced a significant increase in the mean fluorescence intensities (MFIs) of the adhesion molecules CD54 (ICAM-1) and CD102 (ICAM-2) (Fig. 2a), and supernatant accumulation of the cytokines IL-6 (Fig. 2b) and IL-8 (Additional file 4: Figure S4B). On the other hand, MPs-ICs induced a significant increase in the expression of CD54 in microvascular cells from lung HMVEC-L (Fig. 2a), while MPs increased accumulation of the chemokine CCL2 in culture supernatants of these cells (Fig. 2b). Instead, both kinds of vesicles significantly increased accumulation of the chemokine CCL5 in HMVEC-L (Fig. 2b). Nevertheless, vesicles did not have any effect in the endothelial cells from the microvascular dermis HMVEC-D (Fig. 2a and b). Due to the slow growth of HMVEC-D, it was not possible to evaluate the production of cytokines in their culture supernatants (Fig. 2b). MPs and MPs-ICs treatment did not induce accumulation of the other chemokines and cytokines that were evaluated (CCL3, CX3CL1, IL-10, IL-12p70, IL-1β, and TNF-α); also, these soluble factors were not increased when endothelial cells were treated with LPS for 24 h - only accumulation of IL-1β in supernatants was observed after treatment with LPS in HUVEC and HMVEC-L (Additional file 4: Figure S4A). In addition, these vesicles did not have an effect on the expression of HLA-DR in endothelial cells from the macrovasculature and microvasculature (Fig. 2a). These results showed that MPs and MPs-CIs activated endothelial cells and increased their expression of adhesion molecules and some soluble factors. However, this response was heterogeneous depending on the cell origin.

Since we have previously observed an increased count of circulating MPs in patients with SLE [33] and RA [34], we evaluated whether this effect over endothelial cells depends on the amount and source of these vesicles. In the present study, prior to evaluation, the amount of circulating microparticles was determined in patients with SLE and RA and in HCs, and it was found that these patients had a significant increase in plasma concentration of MPs and MPs-ICs (Fig. 2c). Therefore, HUVEC were treated with MPs from HCs and with different ratios of MPs and MPs-ICs from patients with RA. MPs from HCs used to the same proportion as the MPs from patients with SLE and RA (three MPs per cell) increased the expression of the adhesion molecule CD54 in HUVEC (Additional file 4: Figure S4C). No differences in the induction of CD54 were observed between vesicles (MPs and MPs-ICs) in patients with RA and SLE (Additional file 4: Figure S4D); consequently, the effect of these extracellular vesicles was studied regardless of the source, and although all experiments were performed independently for MPs and MPs-IC in each disease, in the present paper they are shown together. MPs and MPs-ICs from patients with RA had a dose-dependent effect in HUVEC, in the expression of CD54 and production of IL-6 and IL-8 (Fig. 2d), which suggests that the increase in the amount of these structures observed in circulation of these patients could lead to endothelial activation.

### MPs and MPs-ICs increased the adhesion of classical and non-classical monocytes to endothelial cells from macrovasculature

**Fig. 3:**
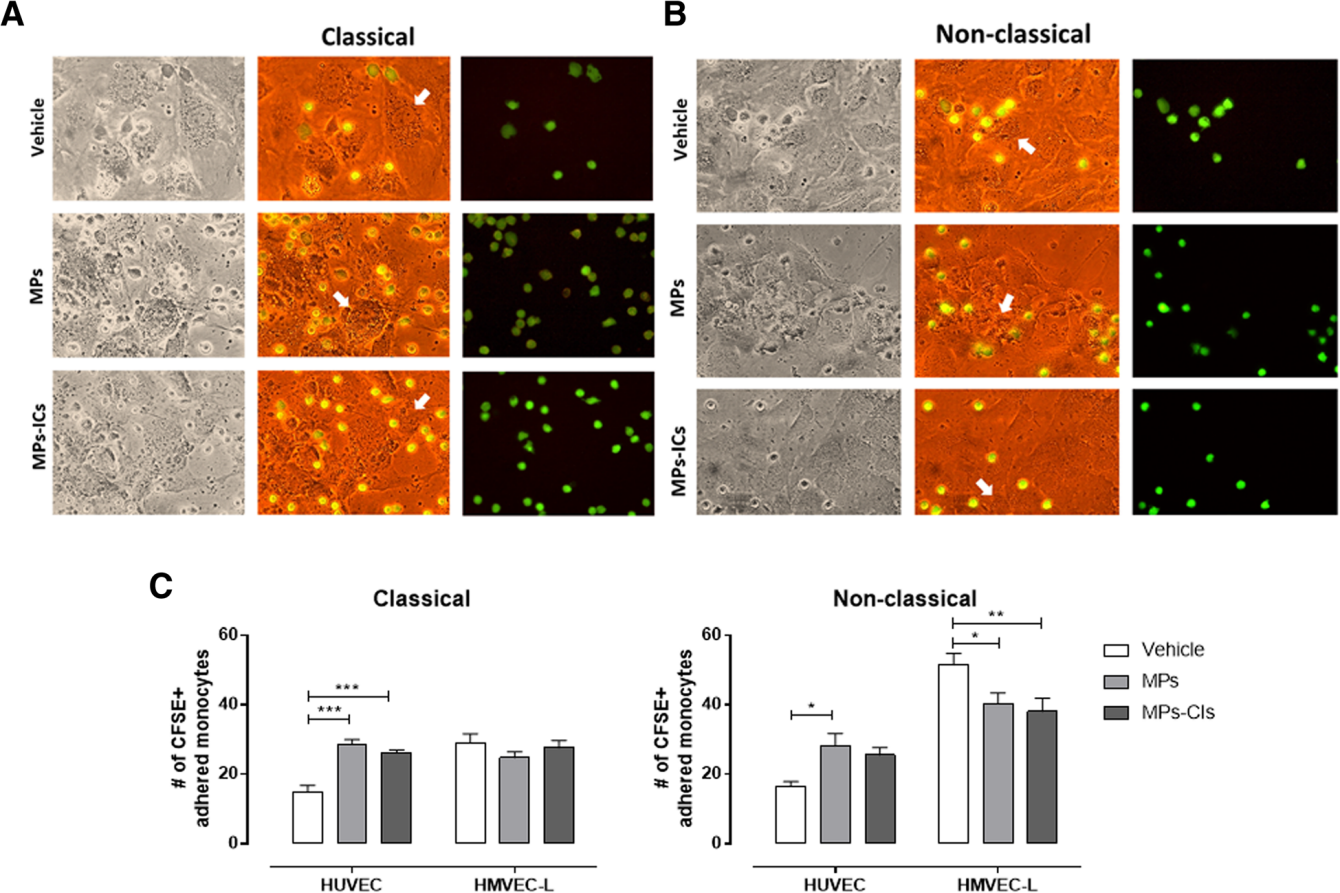
Microparticles (MPs) and microparticles that form immune complexes (MPs-ICs) from patients with systemic lupus erythematosus (SLE) and rheumatoid arthritis (RA) increased the adherence of monocytes to human umbilical vein endothelial cells (HUVEC). Representative pictures of classical (**a**) and non-classical (**b**) monocytes adhered to HUVEC treated with MPs and MPs-ICs from patients with RA and SLE; ×40 objective, arrows indicate the endothelial cell and monocytes are in green. **c** Numbers of classic and non-classical monocytes labeled with carboxyfluorescein succinimidyl ester (CFSE) and adhered to HUVEC or human lung microvascular endothelial cells (HMVEC-L) previously treated with MPs-ICs or MPs from patients with RA and SLE. Data are presented as the mean ± SD. Two-way analysis of variance, *p ≤ 0.05, **p < 0.01, and ***p < 0.001, *n* = 6, monocytes were counted in at least 5 fields by using a ×40 objective

Adhesion of monocytes to the endothelium was evaluated to determine whether the increase in adhesion molecules and chemokine production observed in response to MPs and MPs-ICs has any effect on recruitment of leukocytes [42, 43] (Fig. 3a and b). For HUVEC, MPs and MPs-ICs significantly increased the number of classical monocytes that bound to endothelium (Fig. 3a and c), whereas only MPs induced this interaction for non-classical monocytes (Fig. 3b and c). For HMVEC-L, MPs and MPs-ICs decreased the number of monocyte subsets that adhered to the endothelium. However, in basal conditions, non-classical monocytes interacted mainly with the microvascular (HMVEC-L) cells compared with macrovascular cells (Fig. 3c). These results showed that endothelial cells activated with MPs and MPs-ICs increased the adherence of classical and non-classical monocytes to macrovascular cells but not to endothelial cells in the microvasculature.

### MPs and MPs-ICs induced actin depolymerization and cell–cell adhesion loss in endothelial cells

**Fig. 4:**
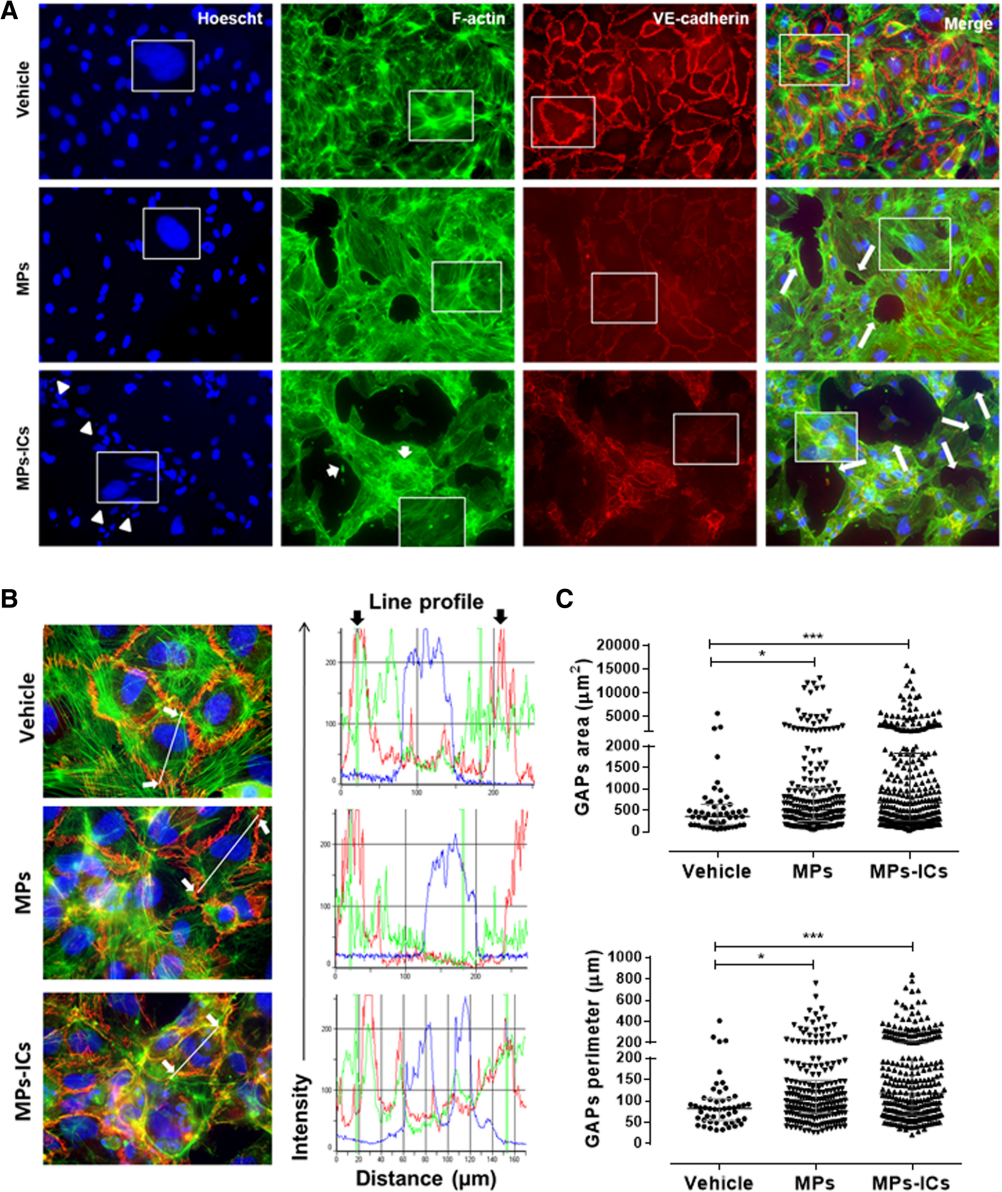
Microparticles (MPs) and microparticles that form immune complexes (MPs-ICs) from patients with systemic lupus erythematosus (SLE) and rheumatoid arthritis (RA) induced intercellular spaces (GAPs) formation, actin depolymerization, and increased expression of VE-cadherin in cytosol. **a** Representative images of the fluorescent labeling of the nucleus (blue), F-actin (actin filaments, in green), VE-cadherin (red), and the superposition of these images (merge) for human umbilical vein endothelial cells (HUVEC) without treatment (vehicle), and treated with MPs or MPs-ICs from patients with RA and SLE for 24 h. Large arrows indicate the presence of GAPs, small arrows indicate actin beads, and arrowheads indicate nuclear condensation and fragmentation; ×20 objective. **b** Fluorescence profile for each label shown in **a**. White arrows indicate the points from which the line was drawn (region of interest (ROI)) to determine the fluorescence profile, and black arrows indicate the plasma membrane of the cells; ×60 objective. **c** GAPs area and perimeter quantification after treatment with MPs and MPs-ICs from patients with RA and SLE. All of the GAPs found in at least 5 fields were measured for each treatment by using a ×20 objective. Data are presented as the median ± interquartile range. Kruskal–Wallis, *p ≤ 0.05, **p < 0.01, ***p < 0.001, *n* = 3

The organization and continuity of the endothelium throughout the vasculature is fundamental for regulation of leukocytes migration, which constitutes a determining factor in the initiation and resolution of inflammatory processes [44]. Therefore, the effect of the MPs and MPs-ICs from patients with RA and SLE in the arrangement of actin filaments and in the cell–cell adhesion in endothelial monolayers of macrovasculature (HUVEC) was evaluated. HUVEC without any treatment (vehicle) presented a normal pattern of polymerization of the actin filaments, forming well-defined stress fibers, and intense expression of VE-cadherin in the plasma membrane that maintains intercellular junctions (Fig. 4a). When the monolayer was treated with MPs, a loss of the continuity in actin filaments and the emergence of GAPs was observed (Fig. 4a) accompanied by a decrease in the membrane expression of VE-cadherin and evident actin depolymerization (Fig. 4a). In addition, the treatment with MPs-ICs seemed to induce more severe changes than treatment with MPs, which showed actin accumulations called “beads” associated with marked depolymerization of actin filaments, a high signal of VE-cadherin in cytosol, and nuclear condensation and fragmentation (Fig. 4a). The fluorescence profiles analysis confirmed these findings (Fig. 4b). Similar results were observed in HMVEC-L treated with MPs and MPs-ICs from patients with RA and SLE (Additional file 5: Figure S5). Actually, both kinds of vesicles significantly increased the area, and perimeter of GAPs in HUVEC monolayers relative to those for cells without any treatment (Fig. 4c), however these changes were more notorious for MPs-ICs. These results showed that MPs and MPs-ICs from patients with RA and SLE altered the structure, organization, and continuity of endothelial monolayers of the macrovasculature and microvasculature.

### MPs and MPs-ICs affected the viability and increased the permeability of the HUVEC monolayer

**Fig. 5:**
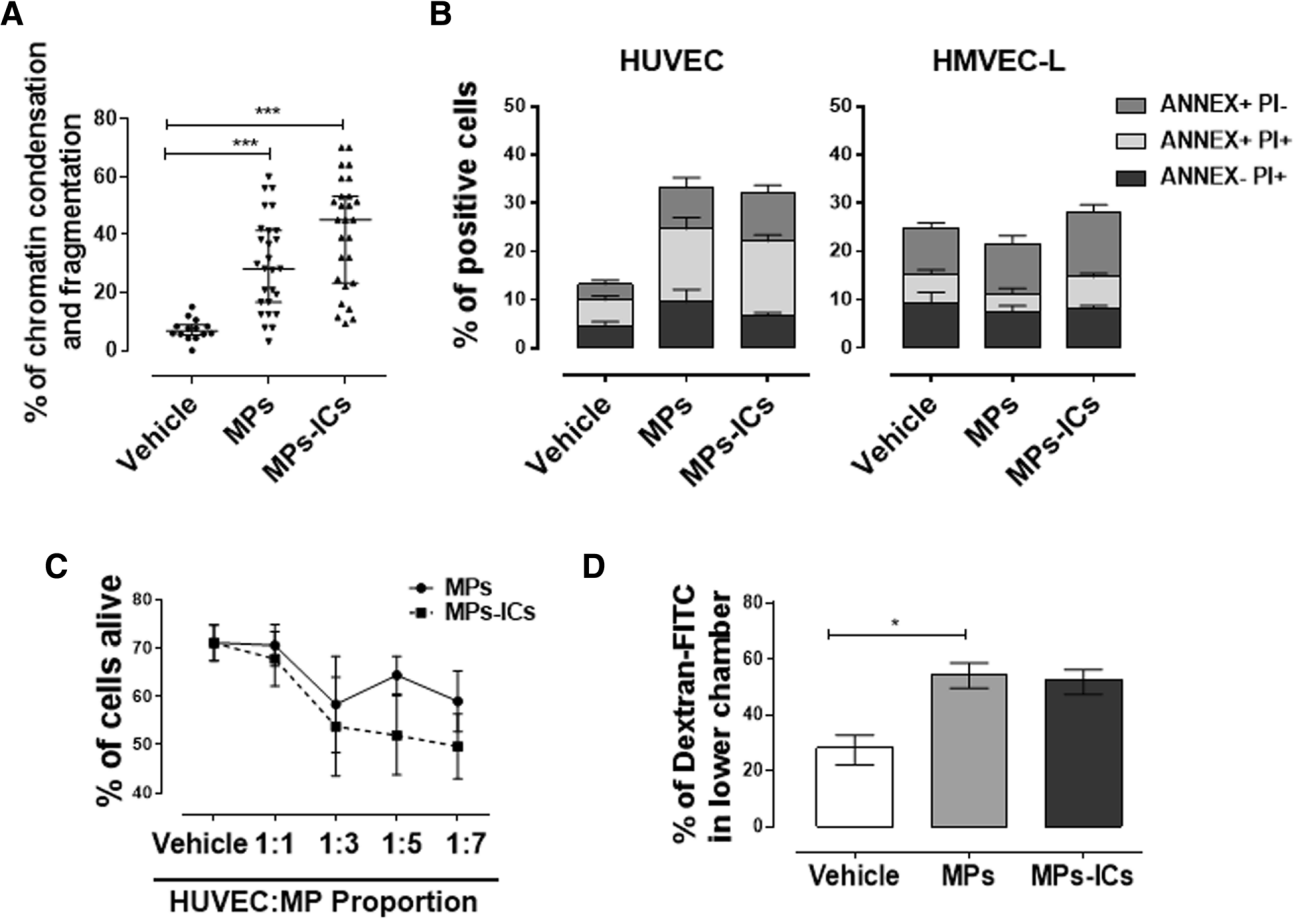
Microparticles (MPs) that form immune complex (MPs-ICs) from patients with systemic lupus erythematosus (SLE) and rheumatoid arthritis (RA) induced cell death and increased the monolayer permeability in human umbilical vein endothelial cells (HUVEC). **a** Percentage of chromatin condensation and fragmentation of HUVEC without treatment (vehicle) or cultured in the presence of MPs or MPs-ICs from patients with RA and SLE for 24 h; data were obtained from ≥ 5 different fields by each experimental condition by using a ×20 objective. Data are presented as the median ± interquartile range. Kruskal–Wallis, *p ≤ 0.05, **p < 0.01, ***p < 0.001, *n* = 3. **b** Percentages of annexin V − PI+ (ANNEX−PI+), annexin V+ PI+ (ANNEX+PI+) and Annexin V− PI+ (ANNEX+PI−) in HUVEC or human lung microvascular endothelial cells (HMVEC-L) after treatment with MPs and MPs-ICs are shown. Data are presented as the mean ± SD. Two-way analysis of variance, *n* = 4. **c** Percentage of living HUVEC after the treatment with different proportions (1:1, 1:3, 1:5, 1:7) of MPs (continuous black line) and MPs-ICs (broken black line) from patients with RA. Data are presented as the median ± interquartile range. Two-way analysis of variance, *n* = 3–6. **d** Percentage of dextran-FITC that crossed the HUVEC monolayer and was detected in the lower chamber after treatment with MPs and MPs-ICs. Data are presented as the median ± interquartile range. Kruskal–Wallis, *p ≤ 0.05, *n* = 4

Because endothelial cell death is one of the key elements in endothelial dysfunction and atherosclerotic plaque progression [38] and considering the observations about nuclear condensation and fragmentation of endothelial cells in the presence of MPs and MPs-CIs, quantification of this phenomenon in HUVEC was performed. MPs and MPs-ICs from patients with RA and SLE significantly increased the percentage of chromatin condensation and fragmentation in endothelial cells of the macrovasculature (Fig. 5a). To verify these results, cell viability was assessed also by using annexin V (ANNEX) and PI. For HUVEC, treatment with MPs and MPs-ICs increased the percentages of ANNEX+PI− and ANNEX+PI+ cells, whereas microvascular HMVEC-L did not have a higher percentage of these populations than those for cells without treatment (Fig. 5b). According to this, viability kinetics showed that the cell death of HUVEC depended on the concentration of MPs and MPs-ICs from patients with SLE and RA (Fig. 5c). These results showed that MPs and MPs-ICs from patients with SLE and RA decreased the viability of HUVEC.

Since MPs and MPs-ICs from patients with RA and SLE induced a loss of the endothelial monolayer continuity and increased cell death of endothelial cells in the macrovasculature, we evaluated whether these alterations were related to a rise in the permeability of the endothelial monolayer. MPs and MPs-ICs increased the permeability of HUVEC to dextran-FITC (Fig. 5d). These results showed that MPs and MPs-ICs from patients with RA and SLE increase the permeability of endothelial cells in the macrovasculature.

## Discussion

This study showed that MPs and MPs-ICs from patients with RA and SLE induced activation of endothelial cells, mainly those in the macrovasculature. This response was evidenced by an increase in the expression of the adhesion molecules CD54 and CD102; the production of inflammatory mediators, such as IL-6, CCL2, and CCL5; and by the adherence of monocytes to these cells. These vesicles also promoted significant changes in the structure of endothelial monolayers, which decreased cell–cell adhesion, depolymerized actin filaments, and triggered cell death; all of these changes may contribute to the increase in endothelial permeability observed in the response to MPs and MPs-ICs. Therefore, our results showed that MPs and MPs-ICs from patients with RA and SLE induced activation and injury of microvascular and especially of macrovascular endothelial cells; this deleterious effect depended on the number of extracellular vesicles that can contact these cells, as the dose–response assays evidenced more dramatic effects with larger amounts of these vesicles.

Extracellular vesicles can take advantage of different routes to interact with their target cells, such as membrane fusion and receptor-mediated endocytosis [36]. Our results showed that MPs, independently of their opsonization, bound to endothelial cells and became internalized. Different endocytic receptors expressed in endothelial cells, such as ICAM-1, scavenger receptor CD36 and receptors for C1q as CD93, can participate in this response [36]. Furthermore, the inhibitory receptor FcγRIIb (CD32), which is expressed in endothelial cells [45], was previously found to be highly endocytic and to participate in the internalization and removal of ICs in a murine model [46]. HUVEC express CD36 and CD32 (Additional file 6: Figure S6), which can be the most probable route of internalization. Therefore, we propose that some of these receptors would mediate endocytosis of MPs and MPs-ICs and induce acidification of endosomes, as observed in the current study. However, with our results we cannot rule out the contribution of other paths through which endothelial cells and extracellular vesicles could interact such as membrane fusion.

Although formation and secretion of MPs are physiological processes, multiple inflammatory and autoimmune diseases, including RA and SLE, involve an increase in the amount of circulating MPs and modifications to the phenotype, such as increased expression of HMGB1, ICs (MPs-ICs), and citrullinated peptides [24, 25, 33]. We have previously detected the presence of HMGB1, citrullinated peptides, and ICs in MPs from patients with SLE [33] and RA [34]. Circulating and urinary levels of HMGB1 have been correlated with disease activity and renal damage in anti-neutrophil cytoplasmic antibody (ANCA)-associated vasculitis (AAV) [47]. The plasma levels of HMGB1 have been found to correlate with endothelial activation in AAV patients (as evaluated by plasma sICAM-1 and VEGF), and HMGB1 to amplify neutrophil activation and glomerular endothelial cells injury, by promoting endothelium-neutrophil interactions [48]. In addition, HMGB1 overexpression (messenger RNA (mRNA) and protein levels) has been associated with an increase in the apoptosis of HUVEC co-cultured with peripheral blood mononuclear cell (PBMC) supernatant from patients with Henoch-Schonlein purpura [49]. These reports suggest that this alarmin can partially induce endothelial activation, phagocytes interaction, and HUVEC apoptosis, which, in the present study, were observed in response to MPs and MPs-ICs from patients with RA and SLE.

Therefore, it is also possible that the increased number of circulating MPs and MPs-ICs in these individuals may further promote contact between endothelial cells and these vesicles, which would induce activation of endothelial cells and change their phenotype. Similar results with different MPs sources have been previously described; for example, Ehsan et al. in 2017 demonstrated that HUVECs showed an increase in the mRNA levels of adhesion molecules VCAM-1, ICAM-1, and chemokine CCL2 after their exposure to MPs from the monocytic cell line THP-1 previously stimulated with LPS [50]. Actually, MPs can modulate different signaling pathways through direct binding of cellular receptors and transfer of a variety of functional macromolecules to the target cells. For example, it has been reported that MPs can contain IL-1β and inflammasome components [22], NF-κβ [51], ICAM-1 [52], and miR-126 [53], among other molecules. All of these mechanisms could explain and contribute to the observed endothelial activation induced by MPs from the patients with RA and SLE in this study.

The increase in the expression of adhesion molecules in endothelial cells and the secretion of the chemokines CCL2 and CCL5 induced by MPs and MPs-ICs from patients with RA and SLE could explain the recruitment and binding of classical monocytes to activated HUVEC. Rautou et al. demonstrated in 2011 that MPs from human atherosclerotic plaques (individuals submitted to endarterectomy) induced expression of ICAM-1 in HUVEC after 24 h of stimulation and promoted subsequent adhesion of U937 promonocytes. This interaction was interrupted by neutralizing antibodies against ICAM-1 and LFA-1 (CD11a/CD18), which indicated that induction of these molecules by the MPs was fundamental for adhesion of these phagocytes [52]. A similar mechanism could be taking place in our case. For microvascular endothelial cells, the induction of adhesion molecules by the MPs was not related to an increase in the adhesion of monocyte subsets. The process of monocyte adhesion to these cells can also be influenced by other molecules, such as E-selectin and P-selectin, CX3CL1, PECAM-1, and CD99, which have previously been identified as involved in monocyte adhesion to the endothelium [54, 55].

The loss of endothelial integrity in autoimmune diseases has been associated with an increase in permeability and recruitment, adhesion, and migration of monocytes to inflamed organs [56]. Disruption of the endothelial barrier in response to MPs has also been observed in other diseases, such as multiple sclerosis and in cerebral ischemia [27, 28]. Marcos-Ramiro et al. in 2014 reported that HUVEC treated with MPs from patients with multiple sclerosis reduced TEER and associated with an increase in the monolayer permeability. Consistent with a decrease in TEER, the formation of GAPs was detected. In addition, these MPs decreased VE-cadherin and ZO-1 expression in the membrane of HCMEC/D3 cells (human brain endothelial cell line) [27]. Similar results were observed with MPs isolated from the plasma of rats (Long–Evans rats) with chronic cerebral ischemia that induced an increase in brain microvascular endothelial cells (RBMVEC) monolayer permeability and DNA fragmentation in a caspase-3-dependent manner. In addition, MPs induced the TNF-α pathway activation since they contained pro-TNF-α, enzyme TACE, and ROCK protein, which were transferred to endothelial cells after recognition [28]. These data and our results suggest that MPs from different sources could directly induce endothelial injury.

Our data showed that HUVEC die in response to MPs and MPs-ICs. This death was characterized by phosphatidylserine exposure and nuclear fragmentation and condensation; therefore, it is possible to suggest that endothelial cells die probably by an apoptotic pathway after vesicle exposition. Edrissi et al. in 2015 also assessed the role of apoptosis in the decrease in TEER and demonstrated that the inhibition of caspase-3 could reverse the increase in TEER caused by MPs [28]. In addition, it was observed that MPs from HCs induce apoptosis but not necrosis, in esophageal and pancreas carcinoma cells by transfer of caspase-3 to target cells [57]. Distler et al. in 2011 also reported that MPs from patients with systemic sclerosis induced the apoptosis of circulating angiogenic cells in a dose-dependent manner [58]. Thus, MPs and MPs-ICs from patients with RA and SLE seem to induce an apoptotic pathway in endothelial cells. However, further studies are required to corroborate this hypothesis.

The mechanisms by which these vesicles induce the endothelial alterations in our case remain to be defined; however, we hypothesize that MPs and MPs-ICs from patients with RA and SLE could induce endothelial injury possibly because of an excessive number of these vesicles that may activate these cells through a variety of signal pathways; no reversal in the monolayer damage was observed with the treatment with atorvastatin and roscovitine drugs and mitochondrial inhibitor metformin (data not shown). The mechanisms that regulate the generation and removal of these vesicles in circulation, rather than a unique and specific effect of these vesicles in endothelial cells, must be a key aspect to control this type of endothelial compromise.

On the other hand, macrovascular and microvascular endothelial cells differ in their response to MPs and MPs-ICs from patients with RA and SLE, as evidenced in this study by the chemokine production, expression of adhesion molecules, susceptibility to damage, and the monocyte subset with which these cells interact. Previously, the differences in phenotype, gene expression, and physiology that exist between macrovascular and microvascular endothelial cells have been described; for example, the amounts of vasoactive substances (endothelin-1, thromboxane, angiotensin II, and prostacyclin) released by these cells have been found to be different after treatment with diverse inducers [59, 60], which demonstrates the importance of establishing the differences in the inflammatory responses according to the kind of vessels from which these cells are derived, but also according to the organs from which these cells originate, as our data showed that the endothelial responses to MPs and MPs-ICs were not the same for the two kinds of microvascular cells evaluated; dermis cells did not respond to these vesicles in contrast to lung cells; therefore, MPs seem to participate in the endothelial injury only in certain organs.

These findings lead us to propose that MPs are important in the endothelial injury of the microvasculature and especially the macrovasculature in the context of RA and SLE. The study of the effect of these vesicles on different endothelial cell types and immune system cells is fundamental for the development of therapeutic strategies that can actually mitigate endothelial injury, decrease comorbidities, and improve the life expectations of patients with RA and SLE.

## Conclusions

Our study shows that MPs and MPs-ICs from patients with RA and SLE mediate activation and injury by macrovascular and microvascular endothelial cells. An excessive amount of these vesicles seems to play an important role in endothelial alterations. Therefore, MPs and MPs-ICs could be an alternative therapeutic target to avoid endothelial injury in these patients.

## Additional files

Additional file 1: **Figure S1.** Strategy of MPs selection and analysis of MPs-ICs percentage. (A) Representative graphs of the strategy of MPs analysis. The desired population was selected using the granularity (SSC A) and size (FSC-A) parameters and the FlowJo V10 program. The percentage of immune complex formation (MPs-CIs) was determined by Overton subtraction (Kolmogorov–Smirnov chi square). The red line indicates unopsonized MPs stained with F(ab)_2_ portion against IgG Fc portion. The blue line indicates MPs opsonized (Ops) with IgG from patients with RA and SLE and stained with the same F(ab)_2_ portion. (B) Percentage of MPs forming ICs with IgG from HCs and patients with RA and SLE. Data are presented as the median ± interquartile range. Kruskal–Wallis, *p-value ≤0.05. (C) Distribution graphs of the percentage of MPs-ICs in two groups of patients, one with RA (*n* = 56) and one with SLE (*n* = 56). Following this normal probability plot, the 75 percentile (P75) of these data was selected as the minimum value to consider that MPs form ICs in this study. (TIF 2365 kb)

Additional file 2: **Figure S2.** The different pools of MPs and MPs-ICs from RA, SLE and HCs have a similar effect in the expression of CD54 in HUVEC. (A) MFI of CD54 in HUVEC treated with two different pools of MPs from HCs and MPs-ICs from RA and SLE. Data are presented as the mean ± SD. Two-way ANOVA, *n* = 4. (TIF 442 kb)

Additional file 3: **Figure S3.** HUVEC, HMVEC-L and HMVEC-D express high levels of CD105, CD31 and VWF in early subcultures. MFI of CD105, CD31 and VWF in HUVEC, HMVEC-L and HMVEC-D cells in different number of subcultures. Data are presented as the mean ± SD. Two-way ANOVA, *n* = 4–6 cultures, *p ≤ 0.05. (TIF 1258 kb)

Additional file 4: **Figure S4.** LPS, MPs and MPs-ICs increase the expression of CD54 and production of CCL2, IL-1β and IL-8 in HUVEC. (A) MFI of CD54 and accumulation of CCL2 and IL-1β in the supernatants of HUVEC and HMVEC-L treated with LPS (100 ng/ml, gray bar) as a positive control for 24 h. Data are presented as the mean ± SD. Two-way ANOVA, *n* = 3. (B) Accumulation of IL-8 in the supernatants of HUVEC and HMVEC-L treated with MPs and MPs-ICs. The white bar corresponds to cells without treatment (vehicle), the light gray bar corresponds to cells treated with MPs, and the dark gray bar corresponds to cells treated with MPs-ICs. Data are presented as the mean ± SD. Two-way ANOVA, *p ≤ 0.05, **p < 0.01, and ***p < 0.001, *n* = 6–8. (C) MFI of CD54 in HUVEC treated with MPs from HCs and patients with SLE and RA for 24 h. Data are presented as the median ± interquartile range. Kruskal–Wallis, n = 4. (D) MFI of CD54 in HUVEC treated with MPs and MPs-ICs from patients with RA and SLE compared with cells without treatment (vehicle, dotted line). Data are presented as the mean ± SD. Two-way ANOVA, *n* = 4. (TIF 2077 kb)

Additional file 5: **Figure S5.** MPs and MPs-ICs induce GAPs formation, actin depolymerization and decrease of VE-cadherin. (A) Representative images of the fluorescent labeling of the nucleus (blue), F-actin (actin filaments, in green), VE-cadherin (red), and the superposition of these markers (merge) for HMVEC-L without treatment (vehicle) and treated with MPs or MPs-ICs from patients with RA and SLE over 24 h. Large arrows indicate the presence of GAPs; ×20 objective. (B) Fluorescence profile for each label mentioned in (A). White arrows indicate the points from which the line was drawn (region of interest (ROI)) to determine the fluorescence profile, and black arrows indicate the plasma membrane of the cells; ×60 objective. (TIF 2941 kb)

Additional file 6: **Figure S6.** HUVEC express CD32 and CD36 in their membranes. Representative histograms of CD32 (left) and CD36 (right) expression in HUVEC. Red line indicates unstained cells and blue line indicates cells stained with the respective antibody. (TIF 1001 kb)

## Abbreviations

ACR: American College of Rheumatology
ANOVA: Analysis of variance
CBA: Cytometric bead array
CFSE: Carboxyfluorescein succinimidyl ester
EBM: Endothelial basal medium
ELISA: Enzyme-linked immunosorbent assay
FBS: Fetal bovine serum
GAPs: Intercellular spaces
HCs: Healthy controls
HMVEC-D: Human dermal microvascular endothelial cells
HMVEC-L: Human lung microvascular endothelial cells
HUSVF: Hospital Universitario San Vicente Fundación
HUVEC: Human umbilical vein endothelial cells
ICs: Immune complexes
IL: Interleukin
LPS: Lipopolysaccharide
MFI: Mean fluorescence intensity
MPs: Microparticles
MPs-ICs: Microparticles that form immune complexes
PBS: Phosphate-buffered saline
PPP: Platelet-poor plasma
RA: Rheumatoid arthritis
RBMVEC: Brain microvascular endothelial cells
ROI: Region of interest
SLE: Systemic lupus erythematosus
TEER: Transendothelial resistance
