# Supplementary figures and images for: Endothelial activation and injury by microparticles in patients with systemic lupus erythematosus and rheumatoid arthritis
Source: Arthritis Res Ther. 2019 Jan 23;21:34. doi: 10.1186/s13075-018-1796-4 (PMC6343289; doi:10.1186/s13075-018-1796-4)

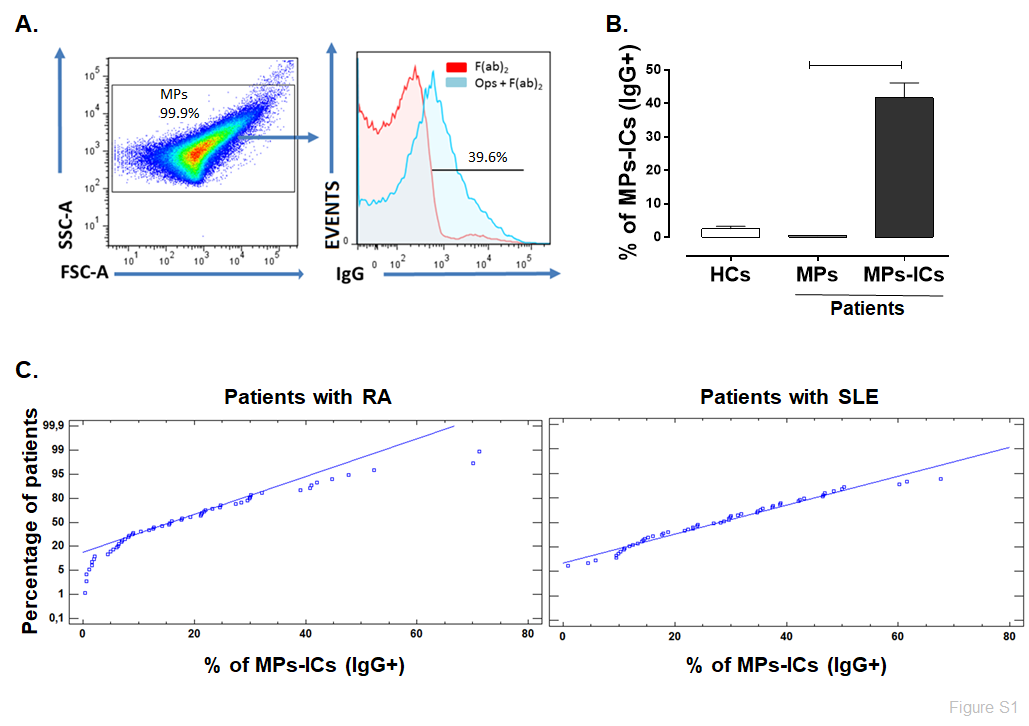

Supplement: Supplementary file 1 — Figure S1. Strategy of MPs selection and analysis of MPs-ICs percentage. (A) Representative graphs of the strategy of MPs analysis. The desired population was selected using the granularity (SSC A) and size (FSC-A) parameters and the FlowJo V10 program. The percentage of immune complex formation (MPs-CIs) was determined by Overton subtraction (Kolmogorov–Smirnov chi square). The red line indicates unopsonized MPs stained with F(ab)2 portion against IgG Fc portion. The blue line indicates MPs opsonized (Ops) with IgG from patients with RA and SLE and stained with the same F(ab)2 portion. (B) Percentage of MPs forming ICs with IgG from HCs and patients with RA and SLE. Data are presented as the median ± interquartile range. Kruskal–Wallis, *p-value ≤0.05. (C) Distribution graphs of the percentage of MPs-ICs in two groups of patients, one with RA (n = 56) and one with SLE (n = 56). Following this normal probability plot, the 75 percentile (P75) of these data was selected as the minimum value to consider that MPs form ICs in this study. (TIF 2365 kb) [file 13075_2018_1796_MOESM1_ESM.tif]

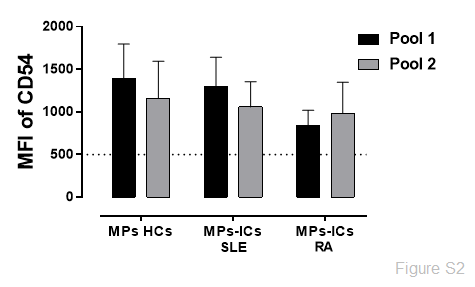

Supplement: Supplementary file 2 — Figure S2. The different pools of MPs and MPs-ICs from RA, SLE and HCs have a similar effect in the expression of CD54 in HUVEC. (A) MFI of CD54 in HUVEC treated with two different pools of MPs from HCs and MPs-ICs from RA and SLE. Data are presented as the mean ± SD. Two-way ANOVA, n = 4. (TIF 442 kb) [file 13075_2018_1796_MOESM2_ESM.tif]

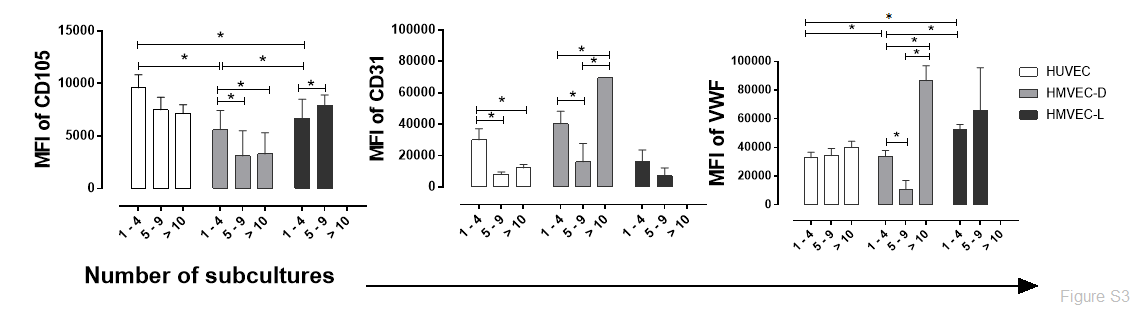

Supplement: Supplementary file 3 — Figure S3. HUVEC, HMVEC-L and HMVEC-D express high levels of CD105, CD31 and VWF in early subcultures. MFI of CD105, CD31 and VWF in HUVEC, HMVEC-L and HMVEC-D cells in different number of subcultures. Data are presented as the mean ± SD. Two-way ANOVA, n = 4–6 cultures, *p ≤ 0.05. (TIF 1258 kb) [file 13075_2018_1796_MOESM3_ESM.tif]

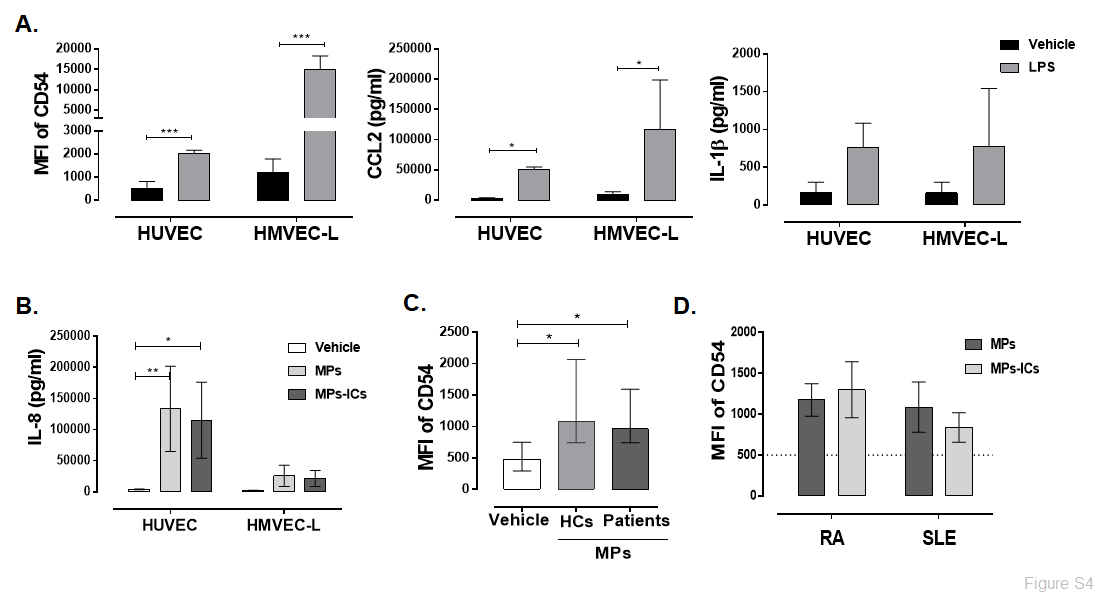

Supplement: Supplementary file 4 — Figure S4. LPS, MPs and MPs-ICs increase the expression of CD54 and production of CCL2, IL-1β and IL-8 in HUVEC. (A) MFI of CD54 and accumulation of CCL2 and IL-1β in the supernatants of HUVEC and HMVEC-L treated with LPS (100 ng/ml, gray bar) as a positive control for 24 h. Data are presented as the mean ± SD. Two-way ANOVA, n = 3. (B) Accumulation of IL-8 in the supernatants of HUVEC and HMVEC-L treated with MPs and MPs-ICs. The white bar corresponds to cells without treatment (vehicle), the light gray bar corresponds to cells treated with MPs, and the dark gray bar corresponds to cells treated with MPs-ICs. Data are presented as the mean ± SD. Two-way ANOVA, *p ≤ 0.05, **p < 0.01, and ***p < 0.001, n = 6–8. (C) MFI of CD54 in HUVEC treated with MPs from HCs and patients with SLE and RA for 24 h. Data are presented as the median ± interquartile range. Kruskal–Wallis, n = 4. (D) MFI of CD54 in HUVEC treated with MPs and MPs-ICs from patients with RA and SLE compared with cells without treatment (vehicle, dotted line). Data are presented as the mean ± SD. Two-way ANOVA, n = 4. (TIF 2077 kb) [file 13075_2018_1796_MOESM4_ESM.tif]

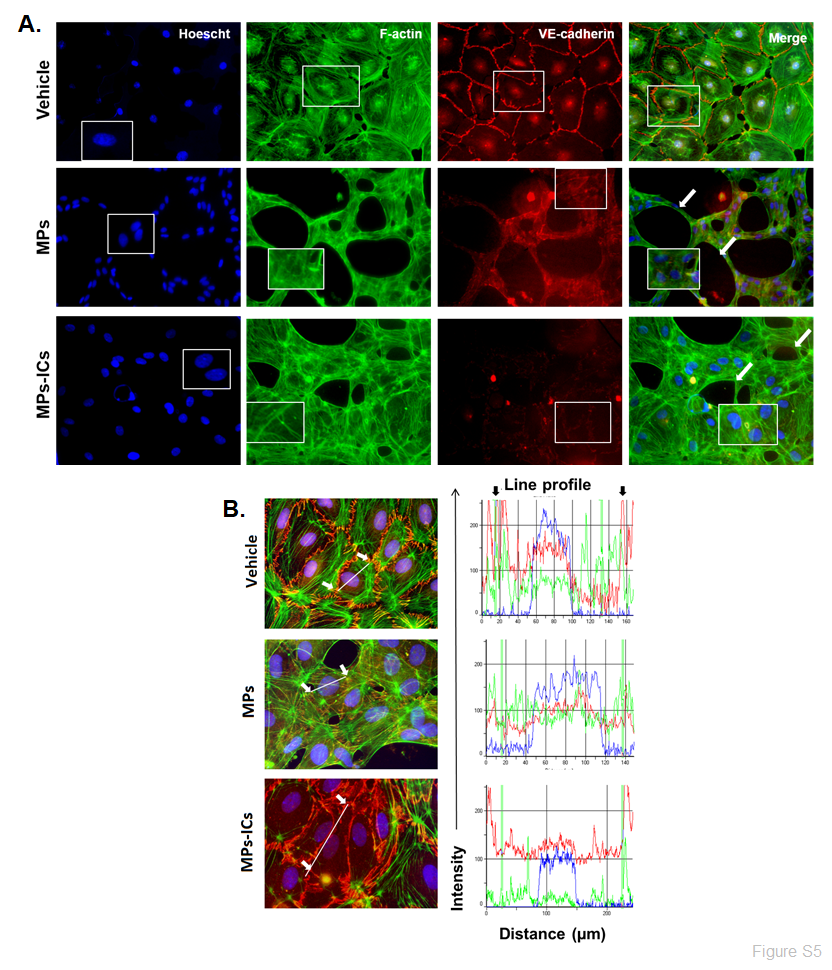

Supplement: Supplementary file 5 — Figure S5. MPs and MPs-ICs induce GAPs formation, actin depolymerization and decrease of VE-cadherin. (A) Representative images of the fluorescent labeling of the nucleus (blue), F-actin (actin filaments, in green), VE-cadherin (red), and the superposition of these markers (merge) for HMVEC-L without treatment (vehicle) and treated with MPs or MPs-ICs from patients with RA and SLE over 24 h. Large arrows indicate the presence of GAPs; ×20 objective. (B) Fluorescence profile for each label mentioned in (A). White arrows indicate the points from which the line was drawn (region of interest (ROI)) to determine the fluorescence profile, and black arrows indicate the plasma membrane of the cells; ×60 objective. (TIF 2941 kb) [file 13075_2018_1796_MOESM5_ESM.tif]

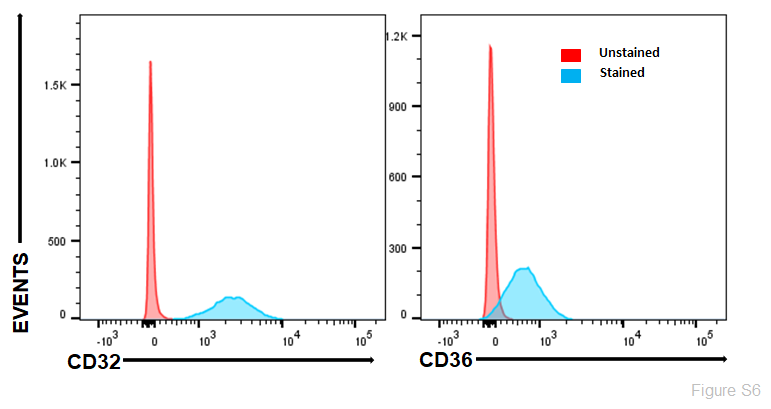

Supplement: Supplementary file 6 — Figure S6. HUVEC express CD32 and CD36 in their membranes. Representative histograms of CD32 (left) and CD36 (right) expression in HUVEC. Red line indicates unstained cells and blue line indicates cells stained with the respective antibody. (TIF 1001 kb) [file 13075_2018_1796_MOESM6_ESM.tif]
